# Nanomechanics of vascular endothelium

**DOI:** 10.1007/s00441-014-1853-5

**Published:** 2014-03-19

**Authors:** Johannes Fels, Pia Jeggle, Ivan Liashkovich, Wladimir Peters, Hans Oberleithner

**Affiliations:** 1 Institute of Cell Dynamics and Imaging, University of Münster, Münster, Germany; 2 Institute of Physiology II, University of Münster, Münster, Germany; 3 Department of Pharmacology, University of Cambridge, Cambridge, UK

**Keywords:** Endothelium, Mechanics, Glycocalyx, Cortex, Nucleus

## Abstract

The mechanical characteristics of endothelial cells reveal four distinct compartments, namely glycocalyx, cell cortex, cytoplasm and nucleus. There is accumulating evidence that endothelial nanomechanics of these individual compartments control vascular physiology. Depending on protein composition, filament formation and interaction with cross-linker proteins, these four compartments determine endothelial stiffness. Structural organization and mechanical properties directly influence physiological processes such as endothelial barrier function, nitric oxide release and gene expression. This review will focus on endothelial nanomechanics and its impact on vascular function.

## Introduction

Endothelial physiology is influenced by numerous biochemical factors. Hormones, paracrines, autocrines and other mediators define the permeability of the endothelial barrier, the anti-thrombotic nature of the endothelial cell surface and endothelium-dependent blood pressure regulation (reviewed in current special issue; and in Libby 2002; Luscher 1990; Landmesser and Drexler 2007; Vierhapper et al. 1990; Wojciak-Stothard and Ridley 2002; Palmer et al. 1987). Endothelial mechanobiology is a young field of research and little is known about mechanics-dependent signaling pathways. This is mainly due to the lack of proper techniques to quantify mechanics in living cells. Over the last decade, however, considerable progress has been made in various techniques, such as atomic force microscopy, laser tweezers, optical trap, pipette aspiration and microrheology. Experimental science is now equipped with a full tool kit facilitating the investigation of cellular mechanics and its physiological relevance (Lee and Lim 2007; Van Vliet et al. 2003). This review will highlight recent advances in the field of endothelial nanomechanics and its impact in endothelial physiology.

## What is meant by “endothelial nanomechanics”?

Mechanobiology of the vascular system can be separated into cell mechanics and mechanical stimuli. On the one hand, external forces like fluid shear stress (FSS), vessel wall tension, vascular hydrostatic pressure and cell–cell contacts determine the mechanical stimuli in the cardiovascular system. These stresses affect endothelial function via mechanotransduction, i.e., activation of mechanosensitive pathways (Tzima 2006; Ando and Yamamoto 2009; Shyu 2009; Johnson et al. 2011). The corresponding mechanosensors exhibit various elements, including mechanosensitive ion channels, adhesion proteins, tyrosine kinase receptors, or caveolae (Liu et al. 2013). Cell mechanics, on the other hand, describes the dynamics of cell (and tissue) elasticity, measured as mechanical stiffness and its impact on endothelial physiology. In more detail, nanomechanics focuses on the mechanical properties of single subcellular compartments (Roduit et al. 2009; Gaboriaud and Dufrene 2007; Kasas and Dietler 2008). The four most prominent and mechanically distinct compartments in the endothelium are (1) the glycocalyx, (2) the cell cortex, (3) the cytoplasm and (4) the nucleus (Kasas et al. 2005; Dahl et al. 2008; Oberleithner et al. 2009, 2011; Martins et al. 2012; Weinbaum et al. 2007). Recently, nanomechanics has come into the focus of research as it turned out that the stiffness of the single cellular compartments has a crucial impact on endothelial cell function. To understand the exact meaning of cell mechanics and its impact upon physiological mechanisms, it is important to define the molecular basis of the nanomechanical properties and to characterize their influence on cellular signaling processes.

## Mechanics of glycocalyx in endothelial function

**Fig. 1:**
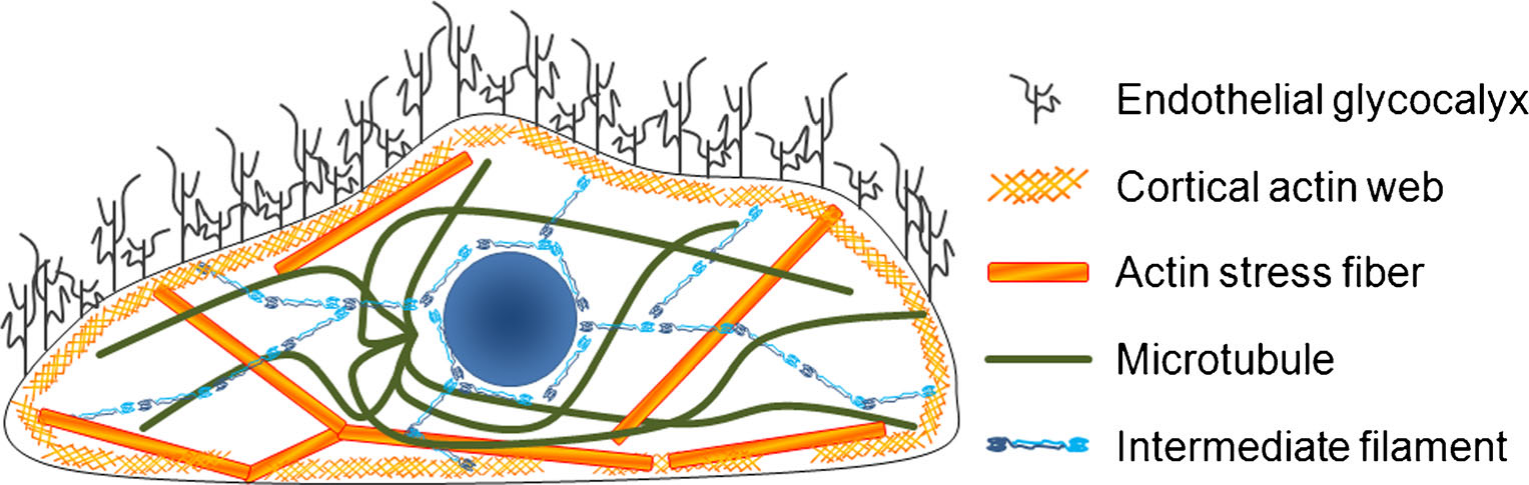
Cellular nanomechanics. Glycocalyx and cytoskeletal organization of endothelial cells determine the mechanical characteristics of the endothelium

The endothelial glycocalyx (eGC) is a thick carbohydrate-rich layer, lining the luminal side of the endothelial surface that consists of proteoglycans and glycoproteins. The proteoglycans are decorated with long carbohydrate side chains, the glycosaminoglycans, among which heparan sulfate is the most prominent in the eGC. This mesh serves as a host for specific plasma proteins, soluble proteoglycans and hyaluronic acid. Together, they form a dynamic and complex interface between blood and tissue (Fig. 1). The total volume of the eGC in the human body is about 1.7 l and its thickness varies from a few hundreds of nanometers in capillaries to a few micrometers in arteries (van den Berg et al. 2003; van Haaren et al. 2003; Nieuwdorp et al. 2006, 2008). Due to its high water content and the loose network, the eGC is several times softer than the underlying subcellular structures (Oberleithner et al. 2011; Peters et al. 2012).

One hallmark function of the eGC is the transmission of biochemical and biomechanical signals from the blood into endothelial cells. Changes in eGC nanomechanics can alter this function (=barrier function). Different processes are known that alter the nanomechanical properties of the eGC. As a polyanionic bio-gel, its volume and mechanics are regulated by the respective electrolyte concentration (Wolf and Gingell 1983; Peters et al. 2012). It has been shown that an extracellular sodium concentration in the upper physiological range leads to a compact eGC (=collapse; Oberleithner et al. 2011). In contrast, treatment of endothelial cells with the polyphenol-rich compound WS1442 induces an increase in volume (=swelling) of the eGC (Peters et al. 2012). The specific mechanisms of collapse and swelling depend on dominant interactions in the system (hydrogen bondings, ionic interactions, hydrophobic/hydrophilic properties, etc.) (Quesada-Perez et al. 2011). Other processes, which modulate the nanomechanics of the eGC, are shedding and biosynthesis. Both can be induced by biochemical factors like hormones and enzymes (Reitsma et al. 2007) or by FSS (Gouverneur et al. 2006; Zeng and Tarbell 2014). Enzymatic digestion of heparan sulfate as well as treatment of endothelial cells with thrombin, lipopolysaccharides, or tumor necrosis factor α compromises the structural integrity of the eGC leading to reduced eGC volume and stiffness (Peters et al. 2012; Wiesinger et al. 2013). In contrast, the biosynthesis of the eGC after enzymatic degradation leads to an increase in volume and a decrease in stiffness, as has been shown in vitro (Bai and Wang 2012).

The eGC is the very first layer of the endothelium that comes into contact with blood. Thus, alterations of eGC nanomechanics lead to a change in the mechanical interaction between blood cells and the eGC constituents. Some theoretical models have been developed that describe such interactions (Weinbaum et al. 2003; Han et al. 2006; Pontrelli et al. 2013). The eGC could serve as a mechanosensor. It is likely that a collapsed eGC can be less deformed by FSS and thereby becomes unable to transmit signals into the cell adequately, a condition that can promote cardiovascular disease. Additional to their function as FSS transmitters, the proteoglycan-associated heparin sulfate residues serve as attachment points for sodium ions and substances like albumin and other blood-borne proteins, hormones and enzymes (Siegel et al. 1996; Reitsma et al. 2007; Quinsey et al. 2004; Kato 2002; Li et al. 1998; Ballinger et al. 2004; Allen et al. 2001; Wilsie and Orlando 2003). Binding of these substrates modifies the nanomechanics of the eGC (Oberleithner et al. 2011; Peters et al. 2012; Job et al. 2012). For instance, increased plasma sodium concentration leads to a stiffening of the glycocalyx and simultaneously increases cellular sodium uptake (Korte et al. 2012; Peters et al. 2012).

**Fig. 2:**
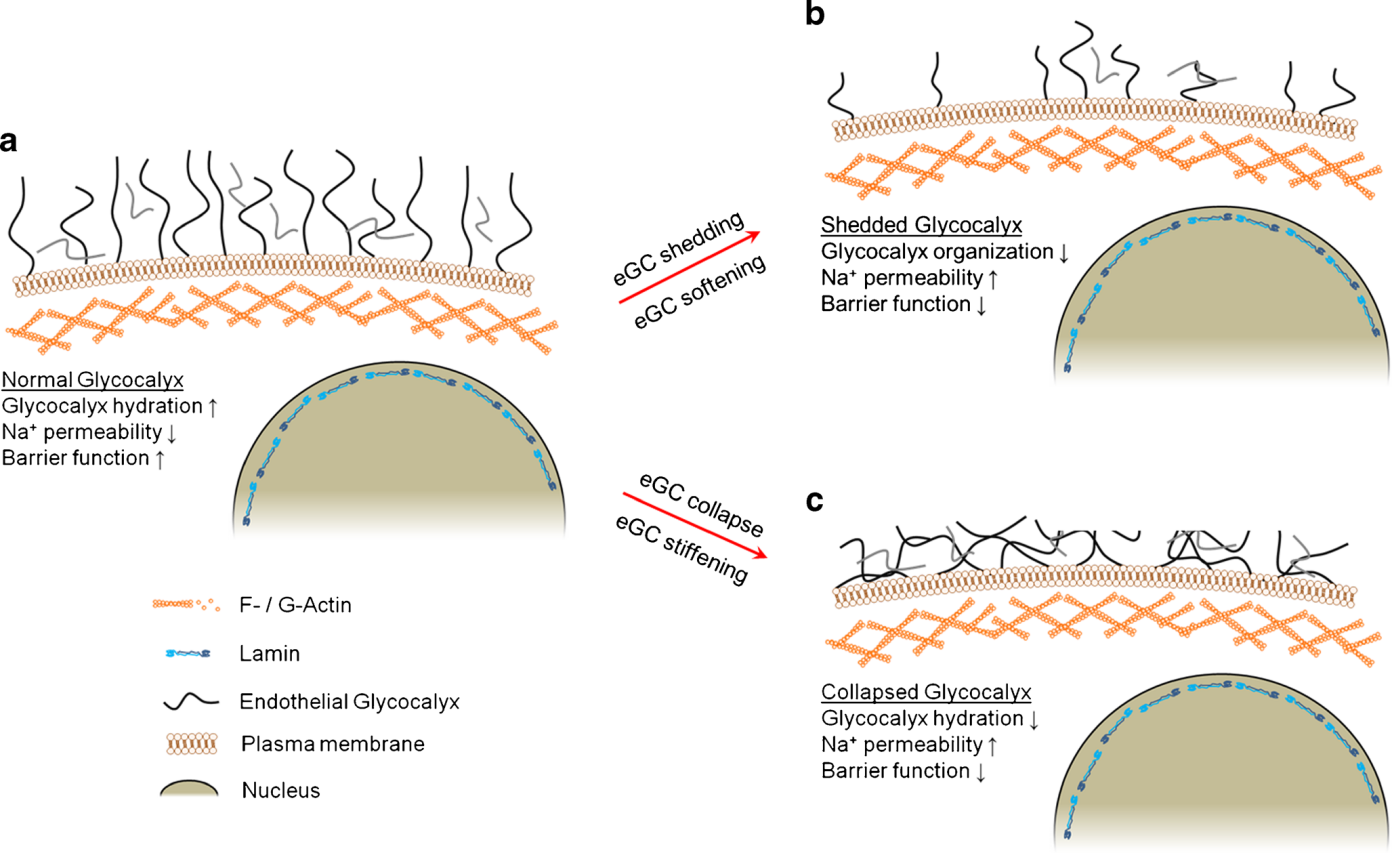
eGC stiffness in endothelial function. **a** A well-hydrated eGC guarantees a functional endothelium. **b** Shedding damages the eGC, which results in decreased barrier function. **c** Acute collapse leads to similar functional changes as described in B but eGC nanomechanics are different

A soft and expanded eGC (Fig. 2a) is supposed to stand for a fully-functional endothelium, whereas a shedded or collapsed eGC most likely exerts adverse effects on the vascular system. Shedding, leading to a reduced eGC with compromised nanomechanics (Fig. 2b), facilitates edema formation (Salmon and Satchell 2012; Strunden et al. 2012; Becker et al. 2010), cell vessel wall interactions (Constantinescu et al. 2003; Henry and Duling 2000; Mulivor and Lipowsky 2004), loss of FSS perception (Thi et al. 2004; Mochizuki et al. 2003) and endothelial dysfunction (Nieuwdorp et al. 2005, 2006; Drake-Holland and Noble 2012). A collapsed (shrunk) eGC (Fig. 2c) has been discussed as being a promoter of vascular diseases (Peters et al. 2012).

## Cortical stiffness in endothelial function

Crossing the cell membrane, the first significant mechanical compartment inside the cell is, directly underneath the plasma membrane, the cell cortex. The biophysical properties of intracellular compartments are mainly determined by cytoskeletal components. Due to the three-dimensional cytoskeletal organization, the cell cortex as well as the cytoplasm and the nucleus, can be characterized by their distinct cytoskeleton-dependent nanomechanical properties (Oberleithner et al. 2009; Kasas et al. 2005; Dahl et al. 2008; Martins et al. 2012). Directly beneath the plasma membrane (50–200 nm), actin is organized in form of a dynamic network (Fig. 1) (Miranda et al. 1974; Koning et al. 2008). The cortical web, also known as peripheral actin, is made of cross-linked actin filaments (F-actin) that provide a supportive structure to the plasma membrane and its embedded proteins (Pollard and Cooper 2009). The organization of the cortical cytoskeleton is highly dynamic as the actin filaments are regulated by a variety of actin binding proteins (dos Remedios et al. 2003). The Arp2/3 complex, activated among others by cortactin, initiates filament formation. Additionally, actin polymerization is stimulated by Cdc42 and Rac1, both members of the Rho GTPase family. Cortactin and other proteins, such as filamin or fascin, stabilize the actin web by cross-linking filaments. Destabilization of the cortical actin network due to filament disassembly is induced by Cofilin, Gelsolin, or RhoA. Finally, motor proteins are able to cross-link actin filaments and simultaneously facilitate the force administration to the cortical web (dos Remedios et al. 2003).

Cortical stiffness is mainly determined by the physiological status of the dynamic submembranous actin web. A high rate of actin polymerization and a dense filament organization go along with a relatively stiff cortex, whereas a depolymerization of F-actin results in cortical softening (Kasas et al. 2005). Filamin A and/or α-actinin cross-link actin filaments and subsequently lead to an increased stiffness of the actin web (Esue et al. 2009; Kasza et al. 2009). Furthermore, motor proteins (e.g., non-muscle myosin II) contribute to cortical stiffness as they generate contractile forces within the filament network. By this, motor proteins induce a lateral tension within the cortical network leading to an inward directed tension (Paluch et al. 2005; Schillers et al. 2010; Stewart et al. 2011; Trepat et al. 2007; Mierke 2011; Gorfinkiel and Blanchard 2011).

There is evidence that nanomechanics of the cell cortex has significant influence on endothelial physiology (Hoffman and Crocker 2009; Paluch et al. 2005; Stewart et al. 2011; Sokolov et al. 2006). In particular, nitric oxide (NO) release and barrier function, both hallmarks of endothelial function, appear to be influenced by cortical nanomechanics.

**Fig. 3:**
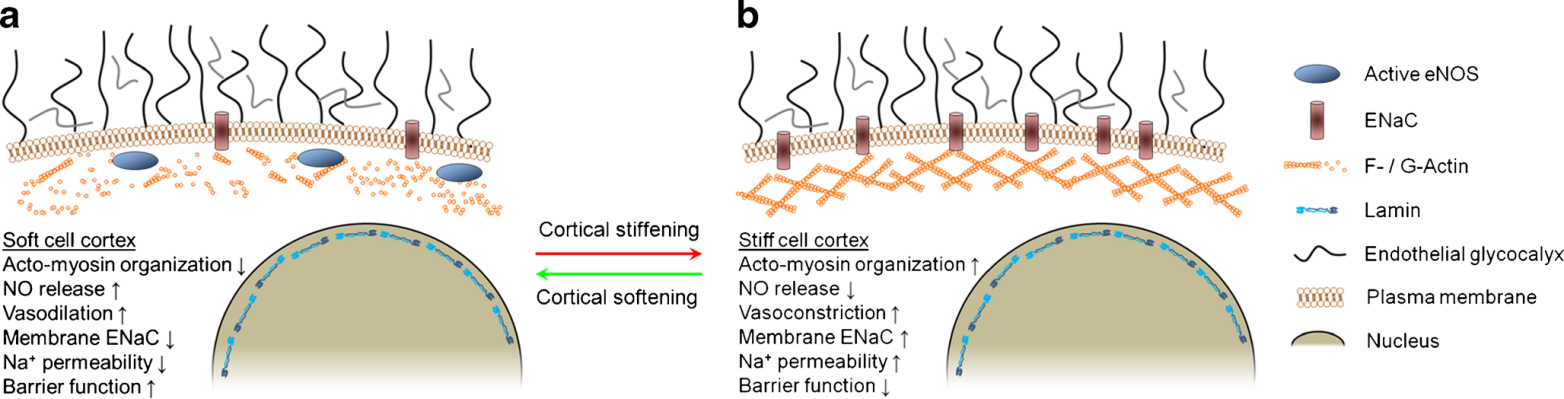
Cortical stiffness influences endothelial function. Softening of the cell cortex, induced by cortical actin depolymerization, decreases membrane abundance of ENaC and endothelial sodium uptake and increases eNOS activity and increases endothelial barrier function

In the vascular endothelium, a softening of the cell cortex induces NO synthesis and thereby is likely to facilitate vasodilation followed by an increase in tissue perfusion and decrease of blood pressure (Oberleithner et al. 2007, 2009; Szczygiel et al. 2011). Under certain physiological conditions, plasma potassium concentration can increase locally up to 12 mM, e.g., due to muscle or neuronal activity (Nordsborg et al. 2003; Kofuji and Newman 2004), which induces a rapid decrease in cortical stiffness (Oberleithner et al. 2009). This softening of the cell cortex is driven by a membrane potential-dependent depolymerization of the submembranous actin web (Callies et al. 2011). A decrease in cortical stiffness is generally caused by a destabilization of the cortical actin web, which verifies the physiological link of elasticity, actin organization and endothelial function (Fels et al. 2012). Simultaneously, the activity of the endothelial NO synthase (eNOS) increases (Fig. 3a). Up to now, there are two mechanisms under discussion by which cortical softening induces eNOS activity. Firstly, it has been shown that eNOS activity is stimulated by an association with G-actin while it is inhibited by an association with F-actin (Kondrikov et al. 2010). A decrease in cortical stiffness due to F-actin depolymerization may increase the association of eNOS with G-actin and therefore directly stimulates NO release (Fels et al. 2012). Secondly, a soft cortex may render the cell more susceptible to shear stress. Mechanosensitive calcium channels in a “flexible” membrane are supposed to be readily activated by shear stress and subsequently increase intracellular calcium levels (Knudsen and Frangos 1997; Kuchan and Frangos 1994; Galan et al. 2011). As eNOS is activated by the calcium-binding protein calmodulin, a soft cortex is likely to promote NO release. Since NO is a vasodilating gas, softening-induced eNOS activity will lead to increased tissue perfusion. Furthermore, blood pressure may decrease in case of systemic softening of the endothelial cortex.

Interestingly, these modulations of cortical stiffness and eNOS activity are independent of the bulk nanomechanics of the endothelial cells (Oberleithner et al. 2009; Fels et al. 2012). While, under physiological conditions, potassium concentrations may rise to a larger extent only locally, other mediators potentially act more systemically, i.e., on the whole vasculature. The mineralocorticoid hormone aldosterone, as well as the cytokine tumor necrosis factor alpha (TNFα), induce transient cortical softening associated with an increase in NO release (Fels et al. 2010; Szczygiel et al. 2011). Interestingly, sustained exposure to aldosterone or TNFα results in an opposite effect, described in detail in the subsequent section. In addition to the regulation of eNOS activity, other vascular mechanisms such as endothelial permeability are influenced by cortical nanomechanics.

Since the link between NO synthesis and cortical stiffness is the dynamic reorganization of the cortical actin cytoskeleton, it was hypothesized that the stiffness-dependent barrier function is again based on cortical actin dynamics.

This hypothesis is verified by the finding that exposure to sphingosine-1-phosphate increases peripheral (cortical) stiffness in pulmonary endothelial cells and thereby increases barrier function. The barrier-enhancing effect is most likely mediated via a signaling cascade including cortactin activation and subsequent actin filament formation in the cell cortex (Arce et al. 2008). In contrast, thrombin acts as the counterpart in regulation of endothelial permeability as it decreases cortactin in the cortex and at the same time increases permeability (Arce et al. 2008). Stimulation of myosin activity, as a second important determinant of cortical stiffness, leads to an increase in barrier function (Dudek et al. 2010). These findings indicate that a decrease in cortical stiffness reduces endothelial barrier function. Hence, it may be concluded that a soft cell cortex indicates a physiological function of the endothelium.

In analogy to cortical softening, increased polymerization of cortical actin and concomitant stiffening of the endothelial cell cortex has (patho-)physiological consequences in the control of endothelial permeability and the response to hormone action (Hall 1984; Birukova et al. 2009). Endothelial stiffening by the C-reactive protein and the cathelicidin LL-37 peptide is found to have anti-inflammatory effects, possibly due to a decrease in endothelial permeability (Kusche-Vihrog et al. 2011; Byfield et al. 2011). These effects contribute to maintaining tissue fluid homeostasis and hence counteract the increased NO production and subsequent drop in blood pressure often accompanying acute inflammatory processes and septic shock. Endothelial cortical stiffening indeed controls endothelial NO release (Fig. 3b), as a cell with a stiff cortex produces reduced amounts of NO (Oberleithner et al. 2007, 2009; Kidoaki and Matsuda 2007; Fels et al. 2010). The phenomenon “increased endothelial cortical stiffness/reduced NO release” was recently termed “stiff endothelial cell syndrome (SECS)” (Lang 2011). Diminished NO release and thus a shift of endothelial action towards increased vasoconstriction, is also the hallmark of endothelial dysfunction, a clinical predictor for expecting cardiovascular diseases later in life (Endemann and Schiffrin 2004; Schachinger et al. 2000). One of the crucial prerequisites leading to an increase in endothelial stiffness and a reduced NO production is a rather high plasma aldosterone level, a major risk factor for vasculopathies. Upon prolonged treatment (>20 min) with aldosterone, endothelial cells swell (Oberleithner et al. 2003; Schneider et al. 2004) and stiffen (Oberleithner et al. 2006). Both of these effects are blocked either by the specific epithelial sodium channel (ENaC) blocker amiloride or by the aldosterone antagonist spironolactone. Both prevent a (further) augmentation in endothelial stiffness upon raising extracellular sodium concentrations from 135 to 145 mM in the presence of aldosterone (Oberleithner et al. 2007). Incidentally, these two factors, aldosterone and high sodium, also cause an increase in ENaC surface expression (Kusche-Vihrog et al. 2008; Korte et al. 2012), indicating a key role of aldosterone in controlling ENaC activity by increasing the channel’s abundance at the endothelial cell surface (Alvarez et al. 2002; Snyder 2002). ENaC is widely abundant in various tissues throughout the human body, including epithelia tissues where this sodium channel mediates the rate-limiting step of sodium transport (Garty and Palmer 1997; Golestaneh et al. 2001). In vascular endothelium, ENaC is different, as the major portion of sodium exits the blood capillary system through a more or less “leaky” paracellular pathway (Mehta and Malik 2006). Also, the ENaC expression level is clearly lower as compared to epithelial tissues (Kusche-Vihrog et al. 2009). Recently, a direct link between ENaC expression and NO release has been established, suggesting the functional role of ENaC in the vascular endothelium (Jeggle et al. 2013). Cells with elevated ENaC expression exhibit an increase in mechanical cortical stiffness in vitro and ex vivo. Taken together, ENaC determines cortical endothelial stiffness and plays a major role in endothelial (dys)function contributing to the control of vascular tone. The mechanistic basis of the link between ENaC and endothelial stiffness most likely relies on the direct interaction of ENaC with F-actin located in the subapical pool underneath the plasma membrane (Mazzochi et al. 2006a, 2006b). Alterations in cortex formation upon changes in the ENaC surface expression could thus also be ascribed to this interaction, as it might increase actin polymerization in this compartment and hence increase cortical stiffness. The sequence of events, whether ENaC membrane insertion promotes actin polymerization or vice versa, has not yet been elucidated.

In addition to chemical mediators, mechanical stimuli affect actin organization (most likely influencing cortical stiffness) and endothelial function. An in vitro increase in hydrostatic pressure, mimicking blood pressure in vivo, induces actin reorganization and affects endothelial permeability (Shin et al. 2003). Fluid shear stress influences actin reorganization (Seebach et al. 2007) and alters the endothelial barrier (Tarbell 2010; Katoh et al. 2008; Ando and Yamamoto 2009; Shyu 2009; Johnson et al. 2011). Finally, substrate stiffness was shown to induce actin polymerization, modulating barrier function in a dose-dependent manner; substrate stiffness simulating “physiological conditions” improves barrier function while stiffer and softer substrates disrupt barrier function (Birukova et al. 2013).

Besides the direct link between cortical stiffness and endothelial function, the elasticity of the cell cortex can be seen as a parameter that determines endothelial physiology in a more general way. For instance, aging cells lose their elasticity due to an increased cytoskeletal organization (Sokolov et al. 2006; Schulze et al. 2010; Kelly et al. 2011; Druppel et al. 2013; Kliche et al. 2011; Qiu et al. 2010). Even basic processes that usually occur in cellular life, such as mitosis, differentiation and development, can be related to changes in cortical stiffness (Stewart et al. 2011; Patel et al. 2012; Kidoaki and Matsuda 2007; Hoffman and Crocker 2009).

One may now assume that a soft cortex is a “fountain of youth” and guarantees a reasonably low blood pressure and a healthy organism. This is, however, not the case. Apparently, the physiological impact of cortical stiffness on physiological mechanisms is highly tissue-specific. In contrast to the endothelium, a soft cortex can even indicate a pathophysiological state of a cell. In ventricular myocytes, for instance, the relationship between elasticity and NO release appears to be different. There, an inhibition of myosin by blebbistatin decreases cell stiffness and simultaneous inhibits NOS activity (Walsh and Cole 2013; Dedkova et al. 2007). Furthermore, it has been shown that the metastatic behavior of cancer cells directly correlates with the cell’s elasticity. Cancer cells with a low elasticity (soft cells) are more likely to spread than stiffer ones (Ketene et al. 2012). Additionally, breast cancer cells induce softening of the endothelium to ease extravasation, leading to facilitated metastasis formation (Mierke 2011).

## Cytoplasm mechanics in endothelial function

While single actin filaments are predominantly found in the cortex, actin stress fibers (bundles of 10–30 filaments) span the whole cytoplasm. These fibers determine cell morphology and stability. They also contribute to focal adhesions and thus are involved in the mechanisms of cell motility (Pellegrin and Mellor 2007; Fletcher and Mullins 2010; Prasain and Stevens 2009). Additionally, the cytoplasm is pervaded by microtubules and intermediate filaments. Microtubules are tube-like polymerized protein (tubulin) filaments facilitating the transport of organelles and vesicles. They are also responsible for the maintenance of cell shape, preventing compression.

Both microtubules and intermediate filaments determine cytoplasmic (bulk) stiffness (Wang 1998; Janmey et al. 1991; Kasas et al. 2005; Herrmann et al. 2007) (Fig. 2).

The molecular nature of the contribution of tubulin to cell nanomechanics has been investigated in detail (Gardel et al. 2008). So far, the microtubule network is supposed to represent a compressive load-bearing component that counteracts the tensile forces generated by the cortical actimyosin web (Ingber 2003).

The physiological relevance in stiffness-dependent signaling pathways in endothelial cells is, however, still unclear. What is known so far is that microtubule-associated proteins (e.g., MAP65) directly influence the flexibility of single microtubules, most likely resulting in a change in cellular elasticity (Portran et al. 2013). Furthermore, in the developing organ of Corti, the fibroblast growth factor induces a microtubule-dependent decrease in cell stiffness leading to hearing loss (Szarama et al. 2012).

Intermediate filaments, of which vimentin is the dominant network-forming member in the endothelium, support the three-dimensional organization of the cell and its organelles (Kamei 1994). Their mechanical properties as well as their contribution to cell mechanics have been reviewed by Herrmann et al. (2007). Intermediate filaments play a key role in mechanotransduction as mutation or deletion of several intermediate filament proteins leads to cell fragility, heart failure and muscle dystrophies. Even leucocyte diapedesis and endothelial nitric oxide release depend on proper intermediate filament formation (Herrmann et al. 2007).

Although the role of intermediate filaments in (endothelial) physiology is well documented and the molecular contributions to cell mechanics are known, evidence for a direct link between intermediate filament-dependent mechanics and endothelial function is still missing. Data on intermediate filament and microtubule mechanics and its impact on endothelial physiology indicate that several different nanomechanical signaling pathways exist that await future investigation.

## Nuclear stiffness in endothelial function

Each individual component of the cellular cytoskeleton described in the previous sections is directly linked to the cell nucleus. Plectin (nesprin-3) provides a link to the cytoplasmic network of intermediate filaments (Wilhelmsen et al. 2005), whereas Nesprin-1/2 mediates binding to microtubules and the actin network (Padmakumar et al. 2004; Zhang et al. 2005). Nesprins connect cytoskeletal networks with the intranuclear lamin network via SUN 1/2 proteins (Haque et al. 2006). It is therefore conceivable that all of the mechanical stimuli perceived by the cell through actin, microtubule, or intermediate filament networks are integrated on the level of the nuclear lamina.

**Fig. 4:**
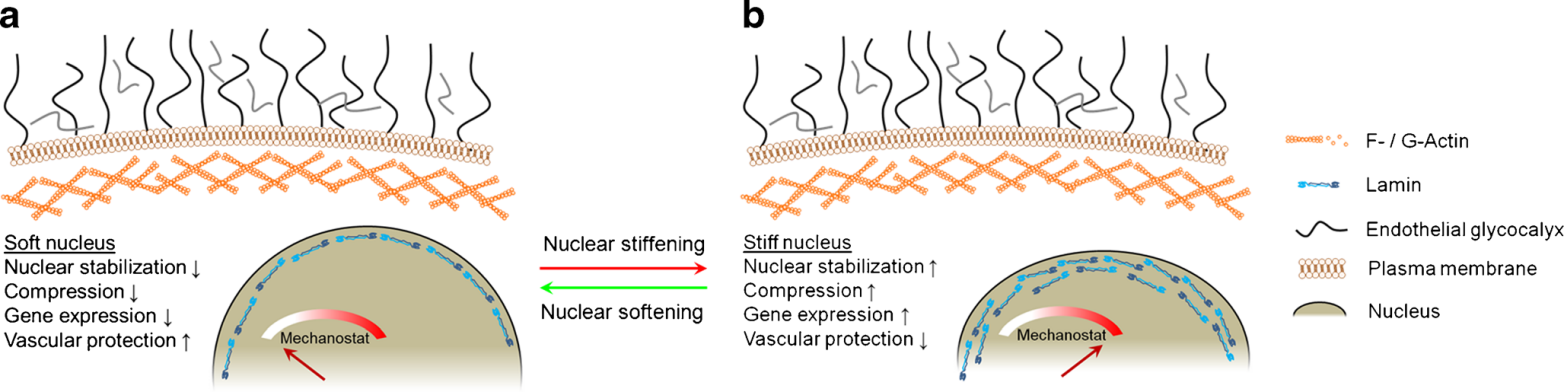
Nuclear elasticity in endothelial function. Elasticity of the cell nucleus regulates gene expression and endothelial function

Similar to the cortical mechanics, the nucleus of an eukaryotic cell could also be an important contributor to the overall mechanics of the cell. Nuclei of several cell types have been shown to be two- to ten-fold stiffer than the respective cytosol (Martins et al. 2012; Ofek et al. 2009). In mechanical terms, the cell nucleus could be envisioned as being a supramolecular shock absorber capable of withstanding considerable stress (Dahl et al. 2004). A connection between cytoskeleton and the nuclear envelope further strengthens the notion of a direct involvement of the nucleus in mechanosensing and mechanotransduction (Maniotis et al. 1997). Recently, the pioneering work from the laboratory of Dennis Discher demonstrated that the nucleus can serve as an intracellular “mechanostat” (Swift et al. 2013), a structure that is able to sense and respond to changes in the mechanical properties of the cellular environment by changing its own stiffness (Fig. 4a).

In the light of recent advances in the field of “nuclear mechanics”, it is tempting to speculate that nuclei of endothelial cells could be directly involved in mediating physiological functions in response to mechanical stimuli. Nuclei of endothelial cells are particularly large in comparison to those of other cell types. Nuclei bulge into the lumina of blood vessels, thus being directly exposed to the shear strain exerted by the blood flow. Consequently, any change in nuclear volume and/or stiffness could have a strong influence on blood flow, in particular in vessels of small inner diameters. According to Hagen-Poisseulle’s law, the flow resistance is related to the 4th potency of the vessel diameter. Thus, a small change in nuclear volume, as shown and quantified in vascular endothelium in response to aldosterone (Oberleithner et al. 2003), could have a considerable impact in arterioles and capillaries. Bulging nuclei could be a significant hindrance for blood flow, in particular when the nuclei stiffen at the same time.

There is evidence that endothelial cell nuclei respond to mechanical stimuli. Nuclei flatten when exposed to physiological shear stress (Deguchi et al. 2005). In addition, nuclei of the cells exposed to shear stress stiffen (Fig. 4b). Apparently, flattening and stiffening of the nuclei could be the consequence of a shape change induced by shear forces. However, the “recovery in shape” and, at the same time, the maintenance of nuclear stiffness upon release of the shear forces, point towards a more complex nuclear remodeling mechanism. The question, whether the expression of the lamin A network at the nucleoplasmic side—a major determinant in the control of the nuclear envelope plasticity (Lammerding et al. 2006)—is responsible for the nuclear ‘mechanostat’ function, remains open (Swift et al. 2013).

There is another reason indicating a potential role of lamin A in the regulation of nuclear plasticity. A mutation of lamin A, which results in an irreversible (permanent) anchoring of the mutated product in the nuclear envelope, results in a severe clinical manifestation termed Hutchison-Gilford progeria syndrome (HGPS) (Merideth et al. 2008). Affected individuals undergo drastically accelerated aging and die in their teens of cardiovascular-related diseases such as stroke or myocardial infarction (Gerhard-Herman et al. 2012). At tissue level, major arteries of HGPS patients demonstrate a severe degeneration of the smooth muscle cell layer (Stehbens et al. 1999). The role of endothelial cells in the development of the syndrome has not been fully assessed to date. However, at the level of individual cell nuclei, the presence of a mutated rigid lamin A network at the inner side of the nuclear envelope has been linked to significant stiffening of such nuclei (Philip and Dahl 2008). It is therefore tempting to speculate that “stiff nuclei” cause an overall stiffening of the endothelial cell. This could result in a severe form of the stiff endothelial cell syndrome (Lang 2011). In this case, the ability of the endothelial cell of sensing and responding to mechanical stimuli might be disrupted. As a result, the downstream signaling directed towards vascular smooth muscle is expected to be impaired, possibly explaining the degradation of vascular smooth muscle cell layer in HGPS.

In summary, the question, whether nuclear mechanics follows mechanical alterations evoked from the exterior environment as postulated by the “nuclear mechanostat” theory (Swift et al. 2013) and/or whether any changes in nuclear elasticity could primarily alter endothelial function, will be an exciting area of cell research in the near future.

## Conclusion and perspectives

In conclusion, the elastic properties of the four compartments, (1) glycocalyx, (2) cortex, (3) cytoplasm and (4) nucleus, are mainly determined by the composition of the respective structural elements. The dynamic interactions between those elements with cross-linker and motor proteins determine the mechanical properties of the respective region. Endothelial nanomechanics has a distinct influence on endothelial function. Various molecular mechanisms control the mechanical properties of living cells. In turn, cellular mechanics control intracellular signaling cascades. Thus, chemical and mechanical signaling pathways are strongly linked to each other. Nanomechanics provides information on the physiological state of the endothelial cell in terms of nitric oxide release and barrier function. A soft cell cortex, combined with a soft, well-hydrated glycocalyx, increases NO formation, which is a prerequisite for a functionally intact vasculature. Any changes in elasticity at the level of the eGC glycocalyx, the cell cortex and the cell nucleus, can have a significant influence on endothelial function in terms of local blood flow, tissue perfusion and, finally, arterial blood pressure.

## References

[CR1] Allen BL, Filla MS, Rapraeger AC (2001). Role of heparan sulfate as a tissue-specific regulator of FGF-4 and FGF receptor recognition. J Cell Biol.

[CR2] Alvarez DLR, Li H, Canessa CM (2002). Effects of aldosterone on biosynthesis, traffic, and functional expression of epithelial sodium channels in A6 cells. J Gen Physiol.

[CR3] Ando J, Yamamoto K (2009). Vascular mechanobiology: endothelial cell responses to fluid shear stress. Circ J.

[CR4] Arce FT, Whitlock JL, Birukova AA, Birukov KG, Arnsdorf MF, Lal R, Garcia JG, Dudek SM (2008). Regulation of the micromechanical properties of pulmonary endothelium by S1P and thrombin: role of cortactin. Biophys J.

[CR5] Bai K, Wang W (2012). Spatio-temporal development of the endothelial glycocalyx layer and its mechanical property in vitro. J R Soc Interface.

[CR6] Ballinger ML, Nigro J, Frontanilla KV, Dart AM, Little PJ (2004). Regulation of glycosaminoglycan structure and atherogenesis. Cell Mol Life Sci.

[CR7] Becker BF, Chappell D, Jacob M (2010). Endothelial glycocalyx and coronary vascular permeability: the fringe benefit. Basic Res Cardiol.

[CR8] Birukova AA, Cokic I, Moldobaeva N, Birukov KG (2009). Paxillin is involved in the differential regulation of endothelial barrier by HGF and VEGF. Am J Respir Cell Mol Biol.

[CR9] Birukova AA, Tian X, Cokic I, Beckham Y, Gardel ML, Birukov KG (2013). Endothelial barrier disruption and recovery is controlled by substrate stiffness. Microvasc Res.

[CR10] Byfield FJ, Wen Q, Leszczynska K, Kulakowska A, Namiot Z, Janmey PA, Bucki R (2011). Cathelicidin LL-37 peptide regulates endothelial cell stiffness and endothelial barrier permeability. Am J Physiol Cell Physiol.

[CR11] Callies C, Fels J, Liashkovich I, Kliche K, Jeggle P, Kusche-Vihrog K, Oberleithner H (2011). Membrane potential depolarization decreases the stiffness of vascular endothelial cells. J Cell Sci.

[CR12] Constantinescu AA, Vink H, Spaan JA (2003). Endothelial cell glycocalyx modulates immobilization of leukocytes at the endothelial surface. Arterioscler Thromb Vasc Biol.

[CR13] Dahl KN, Kahn SM, Wilson KL, Discher DE (2004). The nuclear envelope lamina network has elasticity and a compressibility limit suggestive of a molecular shock absorber. J Cell Sci.

[CR14] Dahl KN, Ribeiro AJ, Lammerding J (2008). Nuclear shape, mechanics, and mechanotransduction. Circ Res.

[CR15] Dedkova EN, Wang YG, Ji X, Blatter LA, Samarel AM, Lipsius SL (2007). Signalling mechanisms in contraction-mediated stimulation of intracellular NO production in cat ventricular myocytes. J Physiol.

[CR16] Deguchi S, Maeda K, Ohashi T, Sato M (2005). Flow-induced hardening of endothelial nucleus as an intracellular stress-bearing organelle. J Biomech.

[CR17] dos Remedios CG, Chhabra D, Kekic M, Dedova IV, Tsubakihara M, Berry DA, Nosworthy NJ (2003). Actin binding proteins: regulation of cytoskeletal microfilaments. Physiol Rev.

[CR18] Drake-Holland AJ, Noble MI (2012). Update on the important new drug target in cardiovascular medicine—the vascular glycocalyx. Cardiovasc Hematol Disord Drug Targets.

[CR19] Druppel V, Kusche-Vihrog K, Grossmann C, Gekle M, Kasprzak B, Brand E, Pavenstadt H, Oberleithner H, Kliche K (2013). Long-term application of the aldosterone antagonist spironolactone prevents stiff endothelial cell syndrome. FASEB J.

[CR20] Dudek SM, Chiang ET, Camp SM, Guo Y, Zhao J, Brown ME, Singleton PA, Wang L, Desai A, Arce FT, Lal R, Van Eyk JE, Imam SZ, Garcia JG (2010). Abl tyrosine kinase phosphorylates nonmuscle Myosin light chain kinase to regulate endothelial barrier function. Mol Biol Cell.

[CR21] Endemann DH, Schiffrin EL (2004). Endothelial dysfunction. J Am Soc Nephrol.

[CR22] Esue O, Tseng Y, Wirtz D (2009). Alpha-actinin and filamin cooperatively enhance the stiffness of actin filament networks. PLoS ONE.

[CR23] Fels J, Callies C, Kusche-Vihrog K, Oberleithner H (2010). Nitric oxide release follows endothelial nanomechanics and not vice versa. Pflugers Arch.

[CR24] Fels J, Jeggle P, Kusche-Vihrog K, Oberleithner H (2012). Cortical actin nanodynamics determines nitric oxide release in vascular endothelium. PLoS ONE.

[CR25] Fletcher DA, Mullins RD (2010). Cell mechanics and the cytoskeleton. Nature.

[CR26] Gaboriaud F, Dufrene YF (2007). Atomic force microscopy of microbial cells: application to nanomechanical properties, surface forces and molecular recognition forces. Colloids Surf B.

[CR27] Galan C, Dionisio N, Smani T, Salido GM, Rosado JA (2011). The cytoskeleton plays a modulatory role in the association between STIM1 and the Ca2+ channel subunits Orai1 and TRPC1. Biochem Pharmacol.

[CR28] Gardel ML, Kasza KE, Brangwynne CP, Liu J, Weitz DA (2008). Chapter 19: Mechanical response of cytoskeletal networks. Methods Cell Biol.

[CR29] Garty H, Palmer LG (1997). Epithelial sodium channels: function, structure, and regulation. Physiol Rev.

[CR30] Gerhard-Herman M, Smoot LB, Wake N, Kieran MW, Kleinman ME, Miller DT, Schwartzman A, Giobbie-Hurder A, Neuberg D, Gordon LB (2012). Mechanisms of premature vascular aging in children with Hutchinson-Gilford progeria syndrome. Hypertension.

[CR31] Golestaneh N, Klein C, Valamanesh F, Suarez G, Agarwal MK, Mirshahi M (2001). Mineralocorticoid receptor-mediated signaling regulates the ion gated sodium channel in vascular endothelial cells and requires an intact cytoskeleton. Biochem Biophys Res Commun.

[CR32] Gorfinkiel N, Blanchard GB (2011). Dynamics of actomyosin contractile activity during epithelial morphogenesis. Curr Opin Cell Biol.

[CR33] Gouverneur M, Berg B, Nieuwdorp M, Stroes E, Vink H (2006). Vasculoprotective properties of the endothelial glycocalyx: effects of fluid shear stress. J Intern Med.

[CR34] Hall PF (1984). The role of the cytoskeleton in hormone action. Can J Biochem Cell Biol.

[CR35] Han Y, Weinbaum S, Spaan JAE, Vink H (2006). Large-deformation analysis of the elastic recoil of fibre layers in a Brinkman medium with application to the endothelial glycocalyx. J Fluid Mech.

[CR36] Haque F, Lloyd DJ, Smallwood DT, Dent CL, Shanahan CM, Fry AM, Trembath RC, Shackleton S (2006). SUN1 interacts with nuclear lamin A and cytoplasmic nesprins to provide a physical connection between the nuclear lamina and the cytoskeleton. Mol Cell Biol.

[CR37] Henry CB, Duling BR (2000). TNF-alpha increases entry of macromolecules into luminal endothelial cell glycocalyx. Am J Physiol Heart Circ Physiol.

[CR38] Herrmann H, Bar H, Kreplak L, Strelkov SV, Aebi U (2007). Intermediate filaments: from cell architecture to nanomechanics. Nat Rev Mol Cell Biol.

[CR39] Hoffman BD, Crocker JC (2009). Cell mechanics: dissecting the physical responses of cells to force. Annu Rev Biomed Eng.

[CR40] Ingber DE (2003). Tensegrity I. Cell structure and hierarchical systems biology. J Cell Sci.

[CR41] Janmey PA, Euteneuer U, Traub P, Schliwa M (1991). Viscoelastic properties of vimentin compared with other filamentous biopolymer networks. J Cell Biol.

[CR42] Jeggle P, Callies C, Tarjus A, Fassot C, Fels J, Oberleithner H, Jaisser F, Kusche-Vihrog K (2013). Epithelial sodium channel stiffens the vascular endothelium in vitro and in liddle mice. Hypertension.

[CR43] Job KM, Dull RO, Hlady V (2012). Use of reflectance interference contrast microscopy to characterize the endothelial glycocalyx stiffness. Am J Physiol Lung Cell Mol Physiol.

[CR44] Johnson BD, Mather KJ, Wallace JP (2011). Mechanotransduction of shear in the endothelium: basic studies and clinical implications. Vasc Med.

[CR45] Kamei H (1994). Relationship of nuclear invaginations to perinuclear rings composed of intermediate filaments in MIA PaCa-2 and some other cells. Cell Struct Funct.

[CR46] Kasas S, Dietler G (2008). Probing nanomechanical properties from biomolecules to living cells. Pflugers Arch.

[CR47] Kasas S, Wang X, Hirling H, Marsault R, Huni B, Yersin A, Regazzi R, Grenningloh G, Riederer B, Forro L, Dietler G, Catsicas S (2005). Superficial and deep changes of cellular mechanical properties following cytoskeleton disassembly. Cell Motil Cytoskeleton.

[CR48] Kasza KE, Nakamura F, Hu S, Kollmannsberger P, Bonakdar N, Fabry B, Stossel TP, Wang N, Weitz DA (2009). Filamin A is essential for active cell stiffening but not passive stiffening under external force. Biophys J.

[CR49] Kato H (2002). Regulation of functions of vascular wall cells by tissue factor pathway inhibitor: basic and clinical aspects. Arterioscler Thromb Vasc Biol.

[CR50] Katoh K, Kano Y, Ookawara S (2008). Role of stress fibers and focal adhesions as a mediator for mechano-signal transduction in endothelial cells in situ. Vasc Health Risk Manag.

[CR51] Kelly GM, Kilpatrick JI, van Es MH, Weafer PP, Prendergast PJ, Jarvis SP (2011). Bone cell elasticity and morphology changes during the cell cycle. J Biomech.

[CR52] Ketene AN, Schmelz EM, Roberts PC, Agah M (2012). The effects of cancer progression on the viscoelasticity of ovarian cell cytoskeleton structures. Nanomedicine.

[CR53] Kidoaki S, Matsuda T (2007). Shape-engineered vascular endothelial cells: nitric oxide production, cell elasticity, and actin cytoskeletal features. J Biomed Mater Res A.

[CR54] Kliche K, Jeggle P, Pavenstadt H, Oberleithner H (2011). Role of cellular mechanics in the function and life span of vascular endothelium. Pflugers Arch.

[CR55] Knudsen HL, Frangos JA (1997). Role of cytoskeleton in shear stress-induced endothelial nitric oxide production. Am J Physiol.

[CR56] Kofuji P, Newman EA (2004). Potassium buffering in the central nervous system. Neuroscience.

[CR57] Kondrikov D, Fonseca FV, Elms S, Fulton D, Black SM, Block ER, Su Y (2010). Beta-actin association with endothelial nitric-oxide synthase modulates nitric oxide and superoxide generation from the enzyme. J Biol Chem.

[CR58] Koning RI, Zovko S, Barcena M, Oostergetel GT, Koerten HK, Galjart N, Koster AJ, Mieke MA (2008). Cryo electron tomography of vitrified fibroblasts: microtubule plus ends in situ. J Struct Biol.

[CR59] Korte S, Wiesinger A, Straeter AS, Peters W, Oberleithner H, Kusche-Vihrog K (2012). Firewall function of the endothelial glycocalyx in the regulation of sodium homeostasis. Pflugers Arch.

[CR60] Kuchan MJ, Frangos JA (1994). Role of calcium and calmodulin in flow-induced nitric oxide production in endothelial cells. Am J Physiol.

[CR61] Kusche-Vihrog K, Callies C, Fels J, Oberleithner H (2009). The epithelial sodium channel (ENaC): Mediator of the aldosterone response in the vascular endothelium?. Steroids.

[CR62] Kusche-Vihrog K, Sobczak K, Bangel N, Wilhelmi M, Nechyporuk-Zloy V, Schwab A, Schillers H, Oberleithner H (2008). Aldosterone and amiloride alter ENaC abundance in vascular endothelium. Pflugers Arch.

[CR63] Kusche-Vihrog K, Urbanova K, Blanque A, Wilhelmi M, Schillers H, Kliche K, Pavenstadt H, Brand E, Oberleithner H (2011). C-reactive protein makes human endothelium stiff and tight. Hypertension.

[CR64] Lammerding J, Fong LG, Ji JY, Reue K, Stewart CL, Young SG, Lee RT (2006). Lamins A and C but not lamin B1 regulate nuclear mechanics. J Biol Chem.

[CR65] Landmesser U, Drexler H (2007). Endothelial function and hypertension. Curr Opin Cardiol.

[CR66] Lang F (2011). Stiff endothelial cell syndrome in vascular inflammation and mineralocorticoid excess. Hypertension.

[CR67] Lee GY, Lim CT (2007). Biomechanics approaches to studying human diseases. Trends Biotechnol.

[CR68] Li Q, Bolli R, Qiu Y, Tang XL, Murphree SS, French BA (1998). Gene therapy with extracellular superoxide dismutase attenuates myocardial stunning in conscious rabbits. Circulation.

[CR69] Libby P (2002). Inflammation in atherosclerosis. Nature.

[CR70] Liu HB, Zhang J, Xin SY, Liu C, Wang CY, Zhao D, Zhang ZR (2013). Mechanosensitive properties in the endothelium and their roles in the regulation of endothelial function. J Cardiovasc Pharmacol.

[CR71] Luscher TF (1990). The endothelium. Target and promoter of hypertension?. Hypertension.

[CR72] Maniotis AJ, Chen CS, Ingber DE (1997). Demonstration of mechanical connections between integrins, cytoskeletal filaments, and nucleoplasm that stabilize nuclear structure. Proc Natl Acad Sci USA.

[CR73] Martins RP, Finan JD, Guilak F, Lee DA (2012). Mechanical regulation of nuclear structure and function. Annu Rev Biomed Eng.

[CR74] Mazzochi C, Benos DJ, Smith PR (2006). Interaction of epithelial ion channels with the actin-based cytoskeleton. Am J Physiol Renal Physiol.

[CR75] Mazzochi C, Bubien JK, Smith PR, Benos DJ (2006). The carboxyl terminus of the alpha-subunit of the amiloride-sensitive epithelial sodium channel binds to F-actin. J Biol Chem.

[CR76] Mehta D, Malik AB (2006). Signaling mechanisms regulating endothelial permeability. Physiol Rev.

[CR77] Merideth MA, Gordon LB, Clauss S, Sachdev V, Smith AC, Perry MB, Brewer CC, Zalewski C, Kim HJ, Solomon B, Brooks BP, Gerber LH, Turner ML, Domingo DL, Hart TC, Graf J, Reynolds JC, Gropman A, Yanovski JA, Gerhard-Herman M, Collins FS, Nabel EG, Cannon RO, Gahl WA, Introne WJ (2008). Phenotype and course of Hutchinson-Gilford progeria syndrome. N Engl J Med.

[CR78] Mierke CT (2011). Cancer cells regulate biomechanical properties of human microvascular endothelial cells. J Biol Chem.

[CR79] Miranda AF, Godman GC, Tanenbaum SW (1974). Action of cytochalasin D on cells of established lines. II. Cortex and microfilaments. J Cell Biol.

[CR80] Mochizuki S, Vink H, Hiramatsu O, Kajita T, Shigeto F, Spaan JA, Kajiya F (2003). Role of hyaluronic acid glycosaminoglycans in shear-induced endothelium-derived nitric oxide release. Am J Physiol Heart Circ Physiol.

[CR81] Mulivor AW, Lipowsky HH (2004). Inflammation- and ischemia-induced shedding of venular glycocalyx. Am J Physiol Heart Circ Physiol.

[CR82] Nieuwdorp M, Meuwese MC, Mooij HL, Ince C, Broekhuizen LN, Kastelein JJ, Stroes ES, Vink H (2008). Measuring endothelial glycocalyx dimensions in humans: a potential novel tool to monitor vascular vulnerability. J Appl Physiol.

[CR83] Nieuwdorp M, Meuwese MC, Vink H, Hoekstra JB, Kastelein JJ, Stroes ES (2005). The endothelial glycocalyx: a potential barrier between health and vascular disease. Curr Opin Lipidol.

[CR84] Nieuwdorp M, van Haeften TW, Gouverneur MC, Mooij HL, van Lieshout MH, Levi M, Meijers JC, Holleman F, Hoekstra JB, Vink H, Kastelein JJ, Stroes ES (2006). Loss of endothelial glycocalyx during acute hyperglycemia coincides with endothelial dysfunction and coagulation activation in vivo. Diabetes.

[CR85] Nordsborg N, Mohr M, Pedersen LD, Nielsen JJ, Langberg H, Bangsbo J (2003). Muscle interstitial potassium kinetics during intense exhaustive exercise: effect of previous arm exercise. Am J Physiol Regul Integr Comp Physiol.

[CR86] Oberleithner H, Callies C, Kusche-Vihrog K, Schillers H, Shahin V, Riethmuller C, MacGregor GA, de Wardener HE (2009). Potassium softens vascular endothelium and increases nitric oxide release. Proc Natl Acad Sci USA.

[CR87] Oberleithner H, Peters W, Kusche-Vihrog K, Korte S, Schillers H, Kliche K, Oberleithner K (2011). Salt overload damages the glycocalyx sodium barrier of vascular endothelium. Pflugers Arch.

[CR88] Oberleithner H, Riethmuller C, Ludwig T, Hausberg M, Schillers H (2006). Aldosterone remodels human endothelium. Acta Physiol (Oxf).

[CR89] Oberleithner H, Riethmuller C, Schillers H, MacGregor GA, de Wardener HE, Hausberg M (2007). Plasma sodium stiffens vascular endothelium and reduces nitric oxide release. Proc Natl Acad Sci USA.

[CR90] Oberleithner H, Schneider SW, Albermann L, Hillebrand U, Ludwig T, Riethmuller C, Shahin V, Schafer C, Schillers H (2003). Endothelial cell swelling by aldosterone. J Membr Biol.

[CR91] Ofek G, Natoli RM, Athanasiou KA (2009). In situ mechanical properties of the chondrocyte cytoplasm and nucleus. J Biomech.

[CR92] Padmakumar VC, Abraham S, Braune S, Noegel AA, Tunggal B, Karakesisoglou I, Korenbaum E (2004). Enaptin, a giant actin-binding protein, is an element of the nuclear membrane and the actin cytoskeleton. Exp Cell Res.

[CR93] Palmer RM, Ferrige AG, Moncada S (1987). Nitric oxide release accounts for the biological activity of endothelium-derived relaxing factor. Nature.

[CR94] Paluch E, Piel M, Prost J, Bornens M, Sykes C (2005). Cortical actomyosin breakage triggers shape oscillations in cells and cell fragments. Biophys J.

[CR95] Patel NR, Bole M, Chen C, Hardin CC, Kho AT, Mih J, Deng L, Butler J, Tschumperlin D, Fredberg JJ, Krishnan R, Koziel H (2012). Cell elasticity determines macrophage function. PLoS ONE.

[CR96] Pellegrin S, Mellor H (2007). Actin stress fibres. J Cell Sci.

[CR97] Peters W, Drueppel V, Kusche-Vihrog K, Schubert C, Oberleithner H (2012). Nanomechanics and sodium permeability of endothelial surface layer modulated by hawthorn extract WS 1442. PLoS ONE.

[CR98] Philip JT, Dahl KN (2008). Nuclear mechanotransduction: response of the lamina to extracellular stress with implications in aging. J Biomech.

[CR99] Pollard TD, Cooper JA (2009). Actin, a central player in cell shape and movement. Science.

[CR100] Pontrelli G, Halliday I, Spencer TJ, Konig CS, Collins MW (2013) Modelling the glycocalyx-endothelium-erythrocyte interaction in the microcirculation: a computational study. Comput Methods Biomech Biomed Engin. Epub ahead of print 10.1080/10255842.2013.799146 23734750

[CR101] Portran D, Zoccoler M, Gaillard J, Stoppin-Mellet V, Neumann E, Arnal I, Martiel JL, Vantard M (2013). MAP65/Ase1 promote microtubule flexibility. Mol Biol Cell.

[CR102] Prasain N, Stevens T (2009). The actin cytoskeleton in endothelial cell phenotypes. Microvasc Res.

[CR103] Qiu H, Zhu Y, Sun Z, Trzeciakowski JP, Gansner M, Depre C, Resuello RR, Natividad FF, Hunter WC, Genin GM, Elson EL, Vatner DE, Meininger GA, Vatner SF (2010). Short communication: vascular smooth muscle cell stiffness as a mechanism for increased aortic stiffness with aging. Circ Res.

[CR104] Quesada-Perez M, Maroto-Centeno JA, Forcada J, Hidalgo-Alvarez R (2011). Gel swelling theories: the classical formalism and recent approaches. Soft Matter.

[CR105] Quinsey NS, Greedy AL, Bottomley SP, Whisstock JC, Pike RN (2004). Antithrombin: in control of coagulation. Int J Biochem Cell Biol.

[CR106] Reitsma S, Slaaf DW, Vink H, van Zandvoort MA, Oude Egbrink MG (2007). The endothelial glycocalyx: composition, functions, and visualization. Pflugers Arch.

[CR107] Roduit C, Sekatski S, Dietler G, Catsicas S, Lafont F, Kasas S (2009). Stiffness tomography by atomic force microscopy. Biophys J.

[CR108] Salmon AH, Satchell SC (2012). Endothelial glycocalyx dysfunction in disease: albuminuria and increased microvascular permeability. J Pathol.

[CR109] Schachinger V, Britten MB, Zeiher AM (2000). Prognostic impact of coronary vasodilator dysfunction on adverse long-term outcome of coronary heart disease. Circulation.

[CR110] Schillers H, Walte M, Urbanova K, Oberleithner H (2010). Real-time monitoring of cell elasticity reveals oscillating myosin activity. Biophys J.

[CR111] Schneider SW, Matzke R, Radmacher M, Oberleithner H (2004). Shape and volume of living aldosterone-sensitive cells imaged with the atomic force microscope. Methods Mol Biol.

[CR112] Schulze C, Wetzel F, Kueper T, Malsen A, Muhr G, Jaspers S, Blatt T, Wittern KP, Wenck H, Kas JA (2010). Stiffening of human skin fibroblasts with age. Biophys J.

[CR113] Seebach J, Donnert G, Kronstein R, Werth S, Wojciak-Stothard B, Falzarano D, Mrowietz C, Hell SW, Schnittler HJ (2007). Regulation of endothelial barrier function during flow-induced conversion to an arterial phenotype. Cardiovasc Res.

[CR114] Shin HY, Bizios R, Gerritsen ME (2003). Cyclic pressure modulates endothelial barrier function. Endothelium.

[CR115] Shyu KG (2009). Cellular and molecular effects of mechanical stretch on vascular cells and cardiac myocytes. Clin Sci (Lond).

[CR116] Siegel G, Walter A, Kauschmann A, Malmsten M, Buddecke E (1996). Anionic biopolymers as blood flow sensors. Biosens Bioelectron.

[CR117] Snyder PM (2002). The epithelial Na + channel: cell surface insertion and retrieval in Na + homeostasis and hypertension. Endocr Rev.

[CR118] Sokolov I, Iyer S, Woodworth CD (2006). Recovery of elasticity of aged human epithelial cells in vitro. Nanomedicine.

[CR119] Stehbens WE, Wakefield SJ, Gilbert-Barness E, Olson RE, Ackerman J (1999). Histological and ultrastructural features of atherosclerosis in progeria. Cardiovasc Pathol.

[CR120] Stewart MP, Helenius J, Toyoda Y, Ramanathan SP, Muller DJ, Hyman AA (2011). Hydrostatic pressure and the actomyosin cortex drive mitotic cell rounding. Nature.

[CR121] Strunden MS, Bornscheuer A, Schuster A, Kiefmann R, Goetz AE, Heckel K (2012). Glycocalyx degradation causes microvascular perfusion failure in the ex vivo perfused mouse lung: hydroxyethyl starch 130/0.4 pretreatment attenuates this response. Shock.

[CR122] Swift J, Ivanovska IL, Buxboim A, Harada T, Dingal PC, Pinter J, Pajerowski JD, Spinler KR, Shin JW, Tewari M, Rehfeldt F, Speicher DW, Discher DE (2013). Nuclear lamin-A scales with tissue stiffness and enhances matrix-directed differentiation. Science.

[CR123] Szarama KB, Stepanyan R, Petralia RS, Gavara N, Frolenkov GI, Kelley MW, Chadwick RS (2012). Fibroblast growth factor receptor 3 regulates microtubule formation and cell surface mechanical properties in the developing organ of Corti. Bioarchitecture.

[CR124] Szczygiel AM, Brzezinka G, Targosz-Korecka M, Chlopicki S, Szymonski M (2011) Elasticity changes anti-correlate with NO production for human endothelial cells stimulated with TNF-alpha. Pflugers Arch 463:487–496 10.1007/s00424-011-1051-1 PMC3276767 22160395

[CR125] Tarbell JM (2010). Shear stress and the endothelial transport barrier. Cardiovasc Res.

[CR126] Thi MM, Tarbell JM, Weinbaum S, Spray DC (2004). The role of the glycocalyx in reorganization of the actin cytoskeleton under fluid shear stress: a “bumper-car” model. Proc Natl Acad Sci USA.

[CR127] Trepat X, Deng L, An SS, Navajas D, Tschumperlin DJ, Gerthoffer WT, Butler JP, Fredberg JJ (2007). Universal physical responses to stretch in the living cell. Nature.

[CR128] Tzima E (2006). Role of small GTPases in endothelial cytoskeletal dynamics and the shear stress response. Circ Res.

[CR129] van den Berg BM, Vink H, Spaan JA (2003). The endothelial glycocalyx protects against myocardial edema. Circ Res.

[CR130] van Haaren PM, VanBavel E, Vink H, Spaan JA (2003). Localization of the permeability barrier to solutes in isolated arteries by confocal microscopy. Am J Physiol Heart Circ Physiol.

[CR131] Van Vliet KJ, Bao G, Suresh S (2003). The biomechanics toolbox: experimental approaches for living cells and biomolecules. Acta Mater.

[CR132] Vierhapper H, Wagner O, Nowotny P, Waldhausl W (1990). Effect of endothelin-1 in man. Circulation.

[CR133] Walsh MP, Cole WC (2013). The role of actin filament dynamics in the myogenic response of cerebral resistance arteries. J Cereb Blood Flow Metab.

[CR134] Wang N (1998). Mechanical interactions among cytoskeletal filaments. Hypertension.

[CR135] Weinbaum S, Tarbell JM, Damiano ER (2007). The structure and function of the endothelial glycocalyx layer. Annu Rev Biomed Eng.

[CR136] Weinbaum S, Zhang X, Han Y, Vink H, Cowin SC (2003). Mechanotransduction and flow across the endothelial glycocalyx. Proc Natl Acad Sci USA.

[CR137] Wiesinger A, Peters W, Chappell D, Kentrup D, Reuter S, Pavenstadt H, Oberleithner HKP (2013). Nanomechanics of the endothelial glycocalyx in experimental sepsis. PLoS ONE.

[CR138] Wilhelmsen K, Litjens SH, Kuikman I, Tshimbalanga N, Janssen H, van DB I, Raymond K, Sonnenberg A (2005). Nesprin-3, a novel outer nuclear membrane protein, associates with the cytoskeletal linker protein plectin. J Cell Biol.

[CR139] Wilsie LC, Orlando RA (2003). The low density lipoprotein receptor-related protein complexes with cell surface heparan sulfate proteoglycans to regulate proteoglycan-mediated lipoprotein catabolism. J Biol Chem.

[CR140] Wojciak-Stothard B, Ridley AJ (2002). Rho GTPases and the regulation of endothelial permeability. Vascul Pharmacol.

[CR141] Wolf H, Gingell D (1983). Conformational response of the glycocalyx to ionic strength and interaction with modified glass surfaces: study of live red cells by interferometry. J Cell Sci.

[CR142] Zeng Y, Tarbell JM (2014). The adaptive remodeling of endothelial glycocalyx in response to fluid shear stress. PLoS ONE.

[CR143] Zhang Q, Ragnauth CD, Skepper JN, Worth NF, Warren DT, Roberts RG, Weissberg PL, Ellis JA, Shanahan CM (2005). Nesprin-2 is a multi-isomeric protein that binds lamin and emerin at the nuclear envelope and forms a subcellular network in skeletal muscle. J Cell Sci.

